# Targeted Breast Milk Fortification for Very Low Birth Weight (VLBW) Infants: Nutritional Intake, Growth Outcome and Body Composition

**DOI:** 10.3390/nu12041156

**Published:** 2020-04-21

**Authors:** Sumesh Parat, Praneeta Raza, May Kamleh, Dennis Super, Sharon Groh-Wargo

**Affiliations:** 1 Department of Pediatrics at MetroHealth Medical Center, Cleveland, OH 44109, USA; dmsuper@gmail.com (D.S.); sgrohwargo@metrohealth.org (S.G.-W.); 2 Department of Pediatrics at Texas Tech University Health Sciences Center, Amarillo, TX 79106, USA; 3 Neurological Institute, Cleveland Clinic, Cleveland, OH 44106, USA; pxc292@case.edu; 4 Health Economics and Outcomes Research, Covance Market Access, Houston, TX 77018, USA; may.kamleh@gmail.com

**Keywords:** targeted milk fortification, body composition, VLBW neonates, human milk, individualized fortification, protein, growth, nutrition

## Abstract

Despite improvements in nutritional management, preterm infants continue to face high rates of postnatal growth restriction. Because variability in breast milk composition may result in protein and energy deficits, targeted fortification has been advocated. We conducted an interventional study to compare body composition and growth outcomes of very low birth weight infants fed targeted protein-fortified human milk (HM) with those fed standard fortified HM. If mother’s own milk was not available, donor milk was used. Weekly analysis of HM with mid-infrared spectroscopy was conducted and additional protein was added to the fortified HM to ensure a protein intake of 4 g/kg/day. Weekly anthropometric measurements were done. Prior to discharge or at 37 weeks, corrected age skinfold thickness (SFT) measurements as well as body composition measurement using air displacement plethysmography were done. Among 36 preterm infants enrolled, those in the targeted group (*n* = 17) received more protein and had a larger flank SFT at study end than those in the standard group (*n* = 19). A pilot post-hoc analysis of subjects having at least 30 intervention days showed a 3% higher fat-free mass in the targeted group. Use of a targeted fortification strategy resulted in a higher protein intake and fat-free mass among those receiving longer intervention.

## 1. Introduction

Preterm infants experience high rates of postnatal growth restriction [1], with a particular need for protein supply for growth and improved neurodevelopmental outcomes [2,3]. Human milk (HM) use is very well tolerated and is acknowledged as ideal nutritional support for improving the survival and long-term outcomes of neonates [4]. HM alone, however, does not provide adequate nutrition for preterm infants owing to increased growth requirements of preterm infants [1] and variability of macronutrient composition (contents of fat, protein and energy) in human milk [4,5,6]. Furthermore, donor milk is considered an adequate substitute in neonatal intensive care unit (NICU) settings when mother’s own milk is not available. Along with the variations in nutritional content between donors and intravariation from a single donor, macronutrient analyses of donor milk have shown that similar to mother’s milk alone, macronutrient constituents in donor milk alone are insufficient to sustain a Very Low Birth Weight (VLBW) infant, with Wojcik et al. [7] citing protein content in donor milk to be only 37% less than that needed for VLBW infants. Fortifying HM at a fixed dose, known as standard fortification, is widely practiced [8], yet poor growth rates have been reported in infants fed standard fortified human milk [4]. Targeted, or individualized, fortification by adjusting for individual macronutrients is associated with better growth outcomes among VLBW infants [5,9,10,11,12,13]. This customized approach can lead to decreased variability in HM macronutrients resulting in improved growth in preterm infants [14]. Still, protein intakes for infants among research reporting different targeted fortification regimens are consistently and significantly lower than recommended values [15,16,17]. Protein is essential for neonatal development, including reaching the expected weight gain. Milk composition analyses show that standard HM fortification especially does not always provide adequate protein intakes for VLBW infants [14,15].

Previous studies assessing targeted fortification have focused on comparing growth outcomes and rates, including weight gain and anthropometric measures, with targeted fortification of protein, fat and carbohydrates. Recent studies, however, have shown that infants supplemented with protein alone had better weight gain and positive nitrogen balance [18,19,20]. In addition, the influence of human milk on fat-free mass accretion in preterm infants has recently been investigated [21]. Yet, few researchers have investigated the influence of targeted fortification on body composition outcomes, which have emerged as a necessary measure of nutrition adequacy [10,14].

Targeted fortification requires a readily available, simple and rapid method for human milk analysis that can be used in NICU settings. Recently, a mid-infrared spectroscopy (MIR) analyzer (MIRIS, Uppsala, Sweden) has been approved for human milk clinical use by the Food and Drug Administration (FDA). Using these spectroscopes, HM fed to infants can be individualized, but this is a labor-intensive process. Previous research has suggested that weekly analysis is likely sufficient to establish an individualized nutritional management plan [22]. A reduced schedule for macronutrient measurement may increase the practical use of MIR analyzers. Thus, examining the effects of targeted fortification is especially important and relevant.

The primary objective of this study was to evaluate the benefits of targeted HM fortification on body composition outcomes of VLBW infants. The secondary objective was to determine the influence of targeted fortification on neonatal growth outcomes. We hypothesized that infants fed protein-targeted fortified HM (targeted group) will have improved body composition outcomes and growth at the time of discharge or reaching term age as compared to those in the standard group.

## 2. Materials and Methods

### 2.1. Study Design and Subjects

A prospective interventional study was conducted in the NICU at MetroHealth Medical Center, Cleveland, Ohio. Preterm infants born between June 2013 and December 2014 with birth weight less than 1500 grams and mothers consenting to provide breast milk were included in the study. Infants with major congenital malformations, infants with medical problems precluding them from having breast milk and mothers with medical problems precluding them from providing milk (substance abuse and on medications that are harmful to infant while breastfed) were excluded from the study. The MetroHealth Institutional Review Board approved this study and a written informed consent was obtained from each mother.

The NICU admission log was consulted, and when infants fulfilling the criteria were admitted, the mothers were approached and informed consent was obtained for enrollment into study. Mothers were approached during their hospital stay once they were stable and able to comprehend the informed consent process. Infants were randomized to either the targeted or standard group in a 1:1 allocation ratio as soon as mother gave consent. Twins were considered as one and allocated to the same group. The allocation was concealed in a sequentially numbered, opaque sealed envelope that was opened once consent was obtained. Once randomized, they were not blinded to either the investigators or the caregivers.

### 2.2. Human Milk Collection and Storage

Every week, the previous 24 h supply of breast milk from mothers whose infants were randomized to targeted group was collected and mixed thoroughly and a representative sample was taken and frozen to be sent for analysis. The following procedure was completed for sampling. The work area identified had a sink, storage area and work surface in a separate room in the NICU adjacent to the room where breast-feeding occurred. The work surface was cleaned with a sterilization cloth (SaniCloth Plus, PDI Inc, NY, USA). Following handwashing, the investigator retrieved the milk from the refrigerator, maintaining universal precautions. The 24-h supply was kept in one large clean plastic bottle to accommodate its entirety, and it was thoroughly mixed by pouring the milk between two plastic mixing bottles five times. A 15 mL aliquot from the thoroughly mixed 24-h supply was transferred into a subject coded and double labeled (top and side) 70 mL food grade polypropylene container (NeoMed, Inc., Woodstock, GA) and placed in a freezer (≤4 ºF). The remaining human milk was repackaged in the same collection containers and refrigerated for patient use. The milk provided by mom for that whole week was labelled and batched together. Donor milk was provided to all subjects (<1500 g) and continued past 100 mL/kg when mother’s own milk was insufficient. Donor milk was obtained from the Human Milk Banking Association of North America (HMBANA) certified—Mother’s Milk Bank of Ohio (Columbus, OH, USA), as regular donor milk (no caloric density requirement). The donor milk was sent for analysis by sending a representative sample from one of the bottles belonging to each batch number of donor milk.

### 2.3. Nutritional Practices

During the hospital stay, all infants were fed per the institutional feeding protocol based on the birthweight. The institutional feeding protocol has been outlined in a supplementary file (Table S1). Infants were given starter parenteral nutrition (PN) (7.5% D and 2.5% amino acid) on Day 1, and individualized PN including lipids were initiated from Day 2 onwards. The preferred feed was expressed breast milk (EBM). Once enteral feeds reached 75 mL/kg/day, lipids were decreased by 50% and finally stopped at feeds of 120 mL/kg/day.

### 2.4. Milk Fortification

The feeding for this study protocol was based on birth weight. For all VLBW infants, once feed volume reached 80 mL/kg, the expressed breast milk was fortified with a generation one Similac Concentrated Liquid (Abbott Nutrition, OH, USA) human milk fortifier (HMF) by adding 1 packet HMF: 50 mL of expressed EBM. At enteral feeds of 100 mL/kg/day, the fortification was further increased to 1 packet HMF: 25 mL of EBM and continued until discharge or term corrected age. When feed volume reached 120–130 mL/kg, TPN was discontinued and targeted fortification was started the next day when feed volume was between 130–150 mL/kg/day.

Milk provided was analyzed weekly. Calais Human Milk Analyzer (Metron Inc, OH, USA), a filter-based MIR analyzer, was used. This analyzer has been shown to have good agreement with standard laboratory tests, and the methodology is described and evaluated elsewhere [23]. That week’s milk was analyzed based on the representative samples of mother’s 24-h milk supplies, which were frozen, labelled and batched together for each week. In the targeted group, Liquid Protein Fortifier (Abbott Nutrition, OH, USA) was used to adjust the protein content based on the milk analysis to provide a protein of 4 g/kg/d. If there was inadequate mother’s milk available, donor milk was fed during that week, and analysis of the donor milk was done similarly and fortification was done accordingly. MIR milk analysis was performed on the premises of the manufacturer, which was located within 10 miles of our medical center. The analyzer was calibrated at the beginning of each analysis day by the technician. The samples were then analyzed twice by the same technician using the MIR analyzer, and an average was taken. The ethic committee code is IRB1201046.

### 2.5. Body Composition Assessment

At the initiation of feeds, anthropometric measurements including weight, length and head circumference were measured in both groups and documented at weekly intervals. At the time of NICU discharge or term corrected gestational age (37 weeks), whichever came first, both groups had body composition measurements taken. Skinfold thickness was done on the flank, subscapular and triceps area (Harpenden Skinfold Caliper, West Sussex, UK). Three measurements were done and an average of the two closest measurements was taken. Percentage fat, absolute fat and lean measurements were measured using air displacement plethysmography (ADP) (with the PeaPod System) (Cosmed, CA, USA). ADP divides body weight into fat mass (FM) and fat free mass (FFM) [24] and has been validated in term infants [25]; the performance appears to be similar in preterm with reasonable reproducibility and modest accuracy [26,27].

The infant was transferred to the research unit in an open crib/incubator one hour after his/her regular feed. If the infant was on oxygen at term corrected age and on nasal cannula flow, a trial off oxygen for 30 min was completed in the NICU. If the infant tolerated the oxygen wean, he/she was taken to the research unit. On transfer, the infant was initially placed on a warmer and his/her heart rate, breathing and oxygenation was monitored continuously. The infant’s clothes were removed, and a wig cap was placed. All the wires connected to the infant were removed, the feeding tube if any was removed and his/her weight was checked. The infant was then placed in the PeaPod and measurements were done twice. Infants were returned back to the NICU for the next feed.

### 2.6. Statistical Analysis

Estimation of a sample size was based on data from a previous study at MetroHealth [28]. A sample size of 20 in each group was sufficient to achieve 80% power to detect a 15% change in FFM from 2.2 to 2.5 (SD 0.3) at 5% significance (Power Analysis and Sample Size Statistical Software 2012). The data are reported using the following descriptive statistics: mean ± standard deviation (SD) for interval, parametric data; media with the interquartile range for nonparametric interval or ordinal data and percentages for nominal data. Parametric interval data were defined as having both the skewness and kurtosis between a −3 and +3. The patients were analyzed according to the group they were randomized to. The following statistical tests were used to show group differences: pooled variance or separate variance t-test depending on the F value of their respective standard deviations, Mann–Whitney U test for nonparametric interval or ordinal data and Chi square or Fishers exact test for nominal data. The data were analyzed using SPSS Statistical Package, Version 24 (IBM Inc., Armonk, NY, USA). Statistical significance was defined a priori as a *p* < 0.05 (two-tail).

## 3. Results

**Figure 1:**
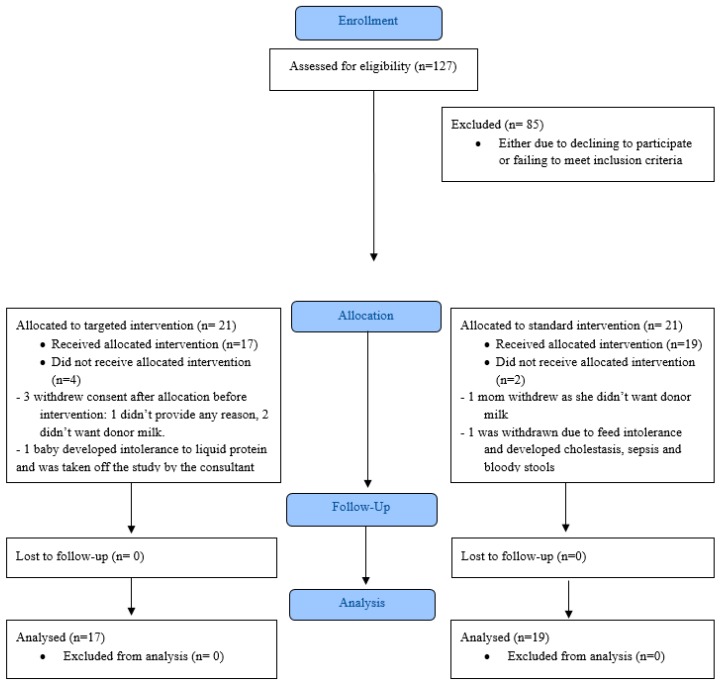
Flowchart of enrollment and participation.

A total of 127 VLBW infants were admitted to MetroHealth’s NICU during the recruitment period from June 2013 to December 2014. In total, 85 VLBW infants were excluded from the study either because they did not meet the inclusion criteria or mothers failed to consent for the study. A flowchart of participant enrollment is shown in Figure 1. A total of 42 infants were randomized. Four mothers (3 in targeted group and 1 in standard group) withdrew from the study after randomization before the intervention was started. One infant in the targeted group developed intolerance to liquid protein and the attending neonatologist decided for discontinuation from the study. Another in the standard group developed sepsis and feeding intolerance and was on prolonged parenteral nutrition with associated cholestasis and was, thus, withdrawn. Thirty-six infants were included in the final analysis.

The clinical characteristics of the infants did not significantly differ between the targeted and standard fortified groups (Table 1). One third of infants in both groups were small for gestational age. Females accounted for 40% of infants in both groups. Almost half of the infants in both groups were African American. There were two sets of twins in both groups.

Gestational age at birth, admission anthropometric data and the growth parameters at start of intervention were comparable in both groups, as seen in Table 1. The average gestational age at birth was 28 weeks, and the intervention was started at around an average of 33 weeks as the unit followed a slow feeding protocol. This resulted in fewer intervention days in most of the subjects before the body composition analyses were taken.

Table 2 presents body composition and growth outcomes for the two groups at the end of the intervention, at term corrected age or at discharge. Among preterm infants fed targeted fortified breast milk, protein intake was significantly higher in the targeted fortification group (4 vs. 3, *p* < 0.001). However, caloric intake was higher in the standard group (*p* = 0.003). Fat mass (*p* = 0.730) was not different between the two groups. Similarly, fat free mass (grams) was not significantly different between the two groups (*p* = 0.066). There were no differences in weight, length, head circumference or mid arm circumference (Table 2). Skin fold thickness showed a statistically significant difference in flank fold thickness (*p* = 0.022) among the targeted fortified group, with no difference in triceps (*p* = 0.980) or subscapular skin fold thickness (*p* = 0.922).

While the majority of the babies had fewer than thirty days of intervention, a pilot post-hoc analysis was completed for infants who received greater than thirty days of intervention (Table 3). There were eight infants in each group. Those in the targeted fortification group had a higher fat free mass % (*p* = 0.046), while fat mass % was higher in the standard group (*p* = 0.046). However, fat free mass (grams) was not statistically different between the two groups (*p* = 0.600). Flank skin fold thickness was higher in the targeted group (*p* = 0.036) with no changes in triceps (*p* = 0.528) and subscapular skinfold thickness (*p* = 0.875).

## 4. Discussion

The is one of the first studies to examine the difference in both body composition and growth outcomes of VLBW infants fed targeted protein-fortified human milk compared to those fed standard fortified human milk. Our findings suggest that targeted fortification results in more protein intake and indicates better body composition growth outcomes compared with standard fortification. Research to date has focused on macronutrient analysis following a targeted fortification protocol [5,17]. In this intervention trial, we were able to perform human milk analysis and adjust protein fortification accordingly to allow infants in the targeted group to receive the recommended protein level of 4 g/kg/day [29].

Along with higher protein intake, the data of pilot post-hoc analysis showed that infants who received at least 30 days of targeted fortified milk exhibited higher fat-free mass than those in the standard fortification group. Previous trials that examined body composition parameters among the targeted preterm infants that were fed have provided mixed results [14,21,30]. McLeod et al. [14] fortified HM using a multi-component human milk fortifier, a protein powder and an energy supplement. They found no significant differences in body composition outcomes compared to infants fed the standard fortification protocol. Morlacchi et al. [11] compared fat-free mass among infants fed protein-fortified human milk with those fed formula and found similar growth in the two groups at discharge, while higher fat-free mass deposition in term-corrected age analyses was shown among infants fed fortified HM. These studies were of shorter duration, compared HM to formula or donor milk, did not focus on protein fortification exclusively or provided lower amounts of fortifier to the milk of intervention infants. While not examined in our study, future research that compares concentrations of essential amino acids between fortification regimens [31,32] may be valuable in understanding the effect of targeted protein fortification on growth outcomes.

On the basis of our findings, targeted fortification of milk with protein can influence the quality of weight gain through promoting fat-free mass in infants. Targeted milk fortification had been evaluated using overall weight gain and anthropometric outcomes [9,10,11,33]. Fat-free mass, however, has recently been established as an important indicator of growth, as greater early gain in fat-free mass, but not fat mass, is associated with improved neurodevelopment in VLBW preterm infants [34]. In a study of 20 preterm infants (gestational age <35 weeks) where researchers assessed weight gain and body composition with respect to measures of cognition up to 4 years, higher percents of fat mass gains were associated with lower-working memory performance [35]. Similarly, a deficit of fat-free mass at discharge was shown to be associated with neurological impairment at two years of age, independent of sex, gestational age and birth weight [36]. Thus, not all weight gain has been correlated with optimal growth. As a result, interventions that target optimal proportionality and quality of weight gain may be more effective for the overall nutritional status and quality of life. Our study presents preliminary data suggesting that intervention through protein targeted fortification may improve these favorable outcomes.

Although the infants in the targeted group in our study did not experience greater weight gain than those in the standard fortification group, higher skin fold thickness was reported amongst those receiving targeted fortified milk. Flank skinfold, weight and length measurements have been validated in an equation that predicts fat mass in term infants [37]. Skinfold measurements in preterm infants, however, are difficult and are not reliably correlated with more accurate methods of body composition measurements, such as ADP [38]. Previous research on the benefits of targeted fortification of all macronutrients has shown inconsistent results [9,10,12]. For instance, Reali et al. [12] showed that the use of targeted fortification led to improved growth rates in a cohort of extremely low birth weight infants in the absence of adverse events. However, Rochow et al. [10] showed similar growth between infants who received targeted fortification and those fed standard fortified milk. Similarly, Mcleod et al. [14] did not find any improvement in growth and nutrition in a group of preterm infants born below 30 weeks of gestation and fed targeted fortification. One possible mitigating reason for these outcomes in our study is that infants who received targeted fortified milk in this study may have received donor milk. Because donor milk is inferior in promoting the growth and development of VLBW preterm infants [39], due to reduced macronutrient content beyond the protein intake, this may explain why infants in the targeted group in this study did not exhibit statistically greater weight gain and caloric intake. Higher caloric intake among the standard group may also be explained by the fact that the milk was not analyzed in the standard fortification group and accepted standard values of calories and protein were used in the calculation; thus, this may lead to over-estimation in the standard group.

While this study is of clinical interest, it has some limitations. First, the number of enrolled infants is small. One reason for this is excluding any VLBW infants with conditions that could negatively interfere with growth. Second, only sixteen infants received the intervention for longer than 30 days. Future studies should aim to investigate body composition outcomes among VLBW infants who receive protein-fortified human milk for a longer time period among a higher sample size. Further, the short duration of the study intervention may limit clinical applicability. We speculate that a longer intervention period may result in more favorable body composition outcomes and thus, future research with a longer interventional study design is needed. Finally, because the investigators were not blinded to the treatments, this may introduce possible bias in data collection and outcome assessment. However, a strength of our study design is that the effect of targeted fortification was assessed throughout the entire stay or until term corrected age, allowing for a thorough study of benefits of targeted fortification.

In conclusion, the findings from this study suggest that adequate protein intake among VLBW infants through targeted milk fortification is associated with better growth outcomes. Among those that received the intervention for more than 30 days, this resulted in greater early fat-free mass deposition, suggesting the beneficial effects of targeted fortification with protein on quality of growth. Implementation of targeted fortification of breast milk could contribute to more favorable growth outcomes in the short-term and possibly to the reduction of the burden of long-term adverse outcomes. This study helps to inform future larger intervention studies that are investigating protein-dense targeted fortification on body composition outcomes. Future research is needed to confirm the results in this study and to better explore the long-term effect of protein fortification of human milk consumption on body composition development in VLBW infants, with particular focus on quality rather than quantity of gain among body composition and growth outcomes.

## Figures and Tables

**Table 1:** Characteristics of infants (*n* = 36) according to the feeding regimen (targeted compared with standard fortification groups) ^1^.

	Standard Group (*n* = 19)	Targeted Group (*n* = 17)	*p*-value *
Female, *n* (%)	8/19 (42.3%)	7/17 (41.2%)	0.955
African American, *n* (%)	10/19 (52.6%)	9/17 (52.9%)	0.179
Multiple births, *n* (%)	4/19 (21.1%)	4/17 (23.5%)	1.000
Small for Gestational age, *n* (%)	6/19 (31.6%)	6/17 (35.3%)	0.965
Gestational age at birth (weeks)	28.6 ± 3.6	28.7 ± 2.9	0.874
Birth Weight (grams)	1016 ±326	1069 ± 346	0.641
Birth Length (cm)	36.1 ± 4.1	36.0 ± 4.8	0.958
Birth Head Circumference (cm)	25.6 ± 3.5	25.3 ± 3.0	0.759
Day of life at intervention	35.4 ± 23.4	32.7 ± 19.2	0.713
Gestational age at intervention (weeks)	33.7 ± 2.1	33.4 ± 2.2	0.675
Weight at intervention (grams)	1447 ±275	1551 ± 272	0.263
Length at intervention (cm)	39.6 ± 3.1	40.3 ± 2.8	0.436
Head circumference at intervention (cm)	27.5 ± 2.2	28.5 ± 2.0	0.176

^1^ Values are either reported as *n* (%) or means ± SDs. * *p*-value > 0.10, (two-tail).

**Table 2:** Nutritional intake and growth outcomes of the targeted and standard fortification groups (*n* = 36) at the end of intervention ^1^.

	Standard Group (*n* = 19)	Targeted Group (*n* = 17)	*p*-Value
Postmenstrual age (weeks) at body composition assessment	37.5 ± 1.74	37.9 ± 2.21	0.633
Days of Intervention	26.5 ± 12.4	31.4 ± 16.7	0.324
Protein intake (gram/kg/day)	3.09 ± 0.33	4.07 ± 0.27	<0.001 **
Calorie intake (kcal/kg/day)	116.7 ± 8.3	107.4 ± 9.2	0.003 **
Weight (grams)	2217 ±422	2368 ± 379	0.268
Length (cm)	43.6 ± 2.0	45.0 ± 2.2	0.065 *
Head circumference (cm)	31.8 ± 1.3	31.9 ± 1.5	0.805
Mid arm circumference (cm)	8.34 ± 0.72	8.61 ± 0.90	0.315
Triceps skin fold thickness (mm)	4.1 ± 0.9	4.0 ± 0.9	0.980
Subscapular skin fold thickness (mm)	4.1 ± 1.0	4.1 ± 1.3	0.922
Flank skin fold thickness (mm)	3.3 ± 1.2	4.4 ± 1.5	0.022 **
Fat free mass (%)	82.8 ± 4.8	83.6 ± 3.2	0.557
Fat mass (grams)	381 ± 142	397 ± 130	0.730
Fat free mass (grams)	1784 ± 230	1940 ± 264	0.066 *

^1^ Values are means ± SDs unless otherwise indicated. ** *p*-value < 0.05 (two-tail); * *p*-value < 0.10.

**Table 3:** Body composition and skinfold thickness in infants who had >30 days of intervention (*n* = 16) ^1^.

	Standard Group (*n* = 8)	Targeted Group (*n* = 8)	*p*-Value
Triceps skin fold thickness (mm)	4.68 ± 0.74	4.41 ± 0.97	0.528
Subscapular skin fold thickness (mm)	4.62 ± 0.92	4.55 ± 1.56	0.875
Flank skin fold thickness (mm)	3.53 ± 1.54	5.00 ± 1.46	0.036 **
Fat Mass (%)	22.0 ± 3.1	18.2 ± 2.9	0.046 **
Fat Free Mass (%)	78.1 ± 3.0	81.8 ± 2.9	0.046 **
Fat Mass (grams)	520 ± 87	473 ± 138	0.600
Fat Free Mass (grams)	1856 ± 232	2067 ± 316	0.093

^1^ Values are means ± SDs unless otherwise indicated. ** *p*-value < 0.05 (two-tail); Mann–Whitney U Test.

## Supplementary Materials

The following are available online at https://www.mdpi.com/2072-6643/12/4/1156/s1, Table S1: “Details of Institutional Feeding Protocol for Very Low Birth Weight infants” has been published online alongside the manuscript.
