# Supplementary material for: Targeted Breast Milk Fortification for Very Low Birth Weight (VLBW) Infants: Nutritional Intake, Growth Outcome and Body Composition
Source: Nutrients. 2020 Apr 21;12(4):1156. doi: 10.3390/nu12041156 (PMC7230830; doi:10.3390/nu12041156)
Supplement: Supplementary file 1 [file nutrients-12-01156-s001.pdf]

Table S1. Details of Institutional Feeding Protocol for Very Low Birth Weight infants.

*MBM=maternal breast milk; DBM=donor breast milk; PF=preterm formula high protein 24 kcal/oz

**Birth weight (g)**	**≤750**	**751-1000**	**1001-1250**	**1251-1500**
**NPO days**	10	7	3	0
**Feeding type**	MBM/DBM/ PF*	MBM/DBM/ PF*	MBM/DBM/ PF*	MBM/PF*
**Trophic feeding**	11-21 DOL: 0.5 mL Q2	8-14 DOL: 1 mL Q2	4-7DOL: 1.5 mL Q2	None
**Initial feeding**	22nd day: 1 mL Q2#	15th day: 1.5 mL Q2#	8th day: 2 mL Q2#	3 mL Q3
***Feeding increase**	0.5 mL/FDG QOD until 100 mL/kg/d	0.5 mL/FDG QD until 100 mL/kg/d	1 mL/FDG QD until 100 mL/kg/d	20 mL/kg/d until 150 mL/kg/d
**At 80 ml/kg/d**	Fortification: MBM: 1 packet HMF to 50 ml DBM: mix 1:1 with PF			
**At 100 mL/kg/d**	Increase 10 mL/kg/QOD until 150 mL/kg; Fortify to 1:25 if MBM available or change to PF	Increase 10 mL/kg/QD until 150 mL/kg; Fortify to 1:25 if MBM available or change to PF	Increase 15 mL/kg/QD until 150 mL/kg; Fortify to 1:25 if MBM available or change to PF	Fortify to 1:25 if MBM available

#*Change to Q3° feedings when infant reaches 1250 g, then increase by 1 mL per feeding per day*
